# External validation and improvement of the scoring system for predicting the prognosis in hepatocellular carcinoma after interventional therapy

**DOI:** 10.3389/fsurg.2023.1045213

**Published:** 2023-03-03

**Authors:** Wenying Qiao, Qi Wang, Tingting Mei, Qi Wang, Wen Wang, Yonghong Zhang

**Affiliations:** ^1^ Interventional Therapy Center for Oncology, Beijing You ‘an Hospital, Capital Medical University, Beijing, China; ^2^ Center for Infectious Diseases, Beijing You ‘an Hospital, Capital Medical University, Beijing, China

**Keywords:** HCC, scoring system, external validation, recurrence, ablation, TACE

## Abstract

**Background:**

Currently, locoregional therapies, such as transarterial chemoembolization (TACE) and ablation, play an important role in the treatment of Hepatocellular carcinoma (HCC). However, an easy-to-use scoring system that predicts recurrence to guide individualized management of HCC with varying risks of recurrence remains an unmet need.

**Methods:**

A total of 483 eligible HCC patients treated by TACE combined with ablation from January 1, 2017, to December 31, 2019, were included in the temporal external validation cohort and then used to explore possibilities for refinement of the original scoring system. We investigated the prognostic value of baseline variables on recurrence-free survival (RFS) using a Cox model and developed the easily applicable YA score. The performances of the original scoring system and YA score were assessed according to discrimination (area under the receiver operating curve [AUROC] and Harrell's concordance index [C-statistic]), calibration (calibration curves), and clinical utility [decision curve analysis (DCA) curves]. Finally, improvement in the ability to predict in the different scoring systems was assessed using the Net Reclassification Index (NRI). The YA score was lastly compared with other prognostic scores.

**Results:**

During the median follow-up period of 35.6 months, 292 patients experienced recurrence. In the validation cohort, the original scoring system exhibited high discrimination (C-statistic: 0.695) and calibration for predicting the prognosis in HCC. To improve the prediction performance, the independent predictors of RFS, including gender, alpha-fetoprotein (AFP) and des-*γ*-carboxyprothrombin (DCP), tumor number, tumor size, albumin-to-prealbumin ratio (APR), and fibrinogen, were incorporated into the YA score, an improved score. Compared to the original scoring system, the YA score has better discrimination (c-statistic: 0.712VS0.695), with outstanding calibration and the clinical net benefit, both in the training and validation cohorts. Moreover, the YA score accurately stratified patients with HCC into low-, intermediate- and high-risk groups of recurrence and mortality and outperformed other prognostic scores.

**Conclusion:**

YA score is associated with recurrence and survival in early- and middle-stage HCC patients receiving local treatment. Such score would be valuable in guiding the monitoring of follow-up and the design of adjuvant treatment trials, providing highly informative data for clinical management decisions.

## Introduction

Hepatocellular carcinoma (HCC), the most common primary liver cancer, is the sixth most common malignancy worldwide and the third leading cause of cancer-related mortality with very high incidences in China. Approximately 72% of HCC occurs in Asia, of which China accounts for 47% (1, 2). Currently, locoregional therapies, such as transarterial chemoembolization (TACE) and ablation, play an important role in the treatment of HCC. Local ablation has become the first-line treatment strategy for patients with early-stage HCC and exhibits similar clinical efficacy to surgical resection (3, 4). TACE, the recommended treatment modality for BCLC stage B or intermediate stage HCC, has been proven to prolong overall survival (OS) and recurrence-free survival (RFS) in HCC patients (5, 6). However, 50% of patients suffer from recurrence within the first 3 years after local treatment (7), ultimately leading to unfavorable prognoses. Therefore, it is critical to identify patients at high risk of recurrence after locoregional treatment and then guide physicians in clinical decision-making and subsequent management.

More recently, several staging or scoring systems for HCC prognosis, including the Barcelona Clinic Liver Cancer (BCLC) stage (8), Child–Turcotte–Pugh (CTP) class (9), tumor-node-metastasis (TNM) stage (10), and albumin-bilirubin (ALBI) grade (11) have been applied to assess the prognosis of HCC patients, while no one is most widely accepted with more accurate prediction ability. Meanwhile, considerable tumor heterogeneity remains among patients with different types of tumors and inherent limitations exist in many staging systems whose effects on the local treatment are also debatable, making it difficult to effectively predict the prognosis of HCC patients based on original traditional staging systems (12). Moreover, the computational complexity of the mathematical model is also a shortcoming that limits its application in clinical work. Thus, an easy-to-use prediction scoring system is urgently needed to guide individualized management of HCC with varying risks of recurrence.

Based on the above background, our team developed a novel scoring system built on account of HCC patients diagnosed between 2015 and 2016 to predict the risk of recurrence after local treatment, and it has achieved a good response in clinical use (13). The patients were stratified into low, intermediate, and high-risk groups of recurrence according to predicted probability, with significant statistical differences in RFS among different subgroups. Although our scoring system has important guiding value for screening outpatients in high-risk relapse risk, it has not been externally validated, which will lead to that miscalibration may occur owing to differences in the cases and situations, resulting in lower utility (14, 15). Besides, with the completion of our clinical database, our center has enough cases for temporal external validation of the scoring system, which may temper overoptimistic expectations of prediction model performance in independent data (16, 17). Hence, we designed this study based on patients in 2017–2019 to externally validate our scoring system, and make further refinement for more accurate prediction performance.

## Methods

### Patients and study design

The patients enrolled in the study were from the Beijing You ‘an Hospital, Capital Medical University. A total of 1,053 patients diagnosed in 2017–2019 were screened and 483 eligible patients were ultimately included in the present study (Supplementary Figure S1). Differing from patients in the training cohort who were screened from January 1, 2015, to December 31, 2016 (13), patients in the temporal external validation cohort were screened from January 1, 2017, to December 31, 2019, with the last follow-up of July 1, 2022. Simultaneously, this cohort is used to explore possibilities for refinement of the existing scoring system.

Inclusion criteria for this study were as follows: (1) age ≥18 years and <75 years; (2) patients treated with TACE sequential ablation; (3) patients achieved complete ablation; (4) complete clinical data. Exclusion criteria included as follows: (1) patients with advanced HCC; (2) history of other malignancies; (3) secondary liver cancer; (4) major surgical treatment before 3 weeks of interventional therapy; (5) patients with autoimmune disease, systemic infection or inflammation. Furthermore, the diagnosis of HCC was established by histologic findings and/or the American Association for the Study of Liver Diseases criteria (18).

The study protocol was approved by the Ethics Committee of Beijing You ‘an Hospital and complied with the requirements of the Declaration of Helsinki. As a retrospective study, the requirement for patient written informed consent was waived.

### Data collection

Baseline clinicopathologic characteristics of patients were collected, including age, gender, demographic indicators (history of hypertension, diabetes mellitus, antiviral treatment, etc.), laboratory parameters (blood routine examination, liver function, coagulation function, and hepatitis virus markers, etc.), tumor burden (tumor number, and tumor size), tumor markers (alpha-fetoprotein [AFP] and des-*γ*-carboxyprothrombin [DCP]), etiology, ablation modalities and the number of ablations, etc.

### Treatment procedures

All patients enrolled were treated with TACE, which was performed by 2 interventional radiologists with at least 5 years of experience. For the procedure, the right femoral artery was cannulated by percutaneous puncture under local anesthesia. Then a super-selective microcatheter was inserted into the supplying artery of the tumor. A mixture of adriamycin and iodine oil was then injected, followed by embolization with gelatin sponge pledgets or polyvinyl alcohol particles. Angiography revealed occlusion of the intratumoral vessels, filling with an embolic agent, and loss of tumor staining, which was considered the end point of embolization.

Local ablation was performed under the guidance of CT or magnetic resonance imaging (MRI) within 2 weeks after TACE. The skin was first thoroughly disinfected and covered with a sterile cloth, after which a local anesthetic was injected and the ablation needle was inserted into the skin. Blood pressure, pulse, respiratory rate, and oxygen saturation were monitored during the procedure. After complete ablation was confirmed, coagulation was performed along the needle tract before the probe was removed to prevent needle tract bleeding. Most importantly, the safe ablation range of 0.5–1.0 cm should be reserved to ensure complete coverage of the tumor and achieve complete ablation.

### Follow-up and evaluation

Patients were followed up in the 1st month after discharge and then once every 3 to 6 months thereafter. The follow-up contents included a blood routine examination, liver function, AFP, and CT/MRI examinations. All patients were routinely followed up until July 1, 2022.

The criteria for recurrence were the same as the preoperative diagnostic criteria (18), and early recurrence was defined as tumor recurrence diagnosed within two years after treatment. The definitions of RFS and OS as well as a treatment after relapse were consistent with the original manuscript (13).

### Statistical analysis

No formal sample size calculation was applied since this was an observational study. Categorical variables are presented as numbers (percentage) and compared using chi-square, ANOVA, or Fisher's exact test, while continuous data are expressed as mean ± standard deviation (SD) and analyzed by Student's t-test or Mann–Whitney U test. Survival curves were plotted by the Kaplan–Meier method and compared by the log-rank test. Moreover, receiver operating characteristics (ROC) analysis was performed to determine the optimal species cutoff.

For the validation of the original scoring system, the area under the receiver operating curve (AUROC) and Harrell's concordance index (C-index) were first used to determine discriminative ability and the corresponding area under the curve (AUC) values for years 1, 2 and 3 years were reported. Meanwhile, the calibration curves at different time points (1, 2, and 3 years) were plotted by bootstrapping with 1,000 resamples to evaluate the performance of the scoring system. Then, 1-, 2-, and 3-year decision curve analysis (DCA) was utilized to investigate the clinical net benefit for decision-making. Finally, improvement in the ability to predict in the different scoring systems was assessed using the Net Reclassification Index (NRI).

Improvement to the original scoring system was first analyzed by univariate analyses, and then all variables with *P* < 0.05 were analyzed using backward stepwise Cox regression which is based on the Akaike information criterion (AIC). Eventually, variables with *P* < 0.05 in multivariable analysis were used in the establishment of the YA score.

In addition, the YA score was compared with other prognostic models, including monocyte-to-lymphocyte ratio (MLR), neutrophil-lymphocyte ratio (NLR), platelet-lymphocyte ratio (PLR), ALBI grade, and platelets-albumin-bilirubin (PALBI) grade. The discrimination of each model was assessed by estimating the AUC at each time point.

All statistical analyses were conducted in R 4.1.2 statistical software (R Foundation for Statistical Computing, Vienna, Austria) and SPSS 26.0 software (SPSS, Chicago, IL, USA). And all statistical tests were performed using a two-sided significance level of 0.05.

## Results

### Baseline characteristics of patients in the validation cohort

The baseline characteristics of patients in the validation cohort are shown in (Table 1). Of those, 400 patients (82.8%) were males and 83 (17.2%) were females. The major etiology was hepatitis B virus (HBV) infection, 431 patients (89.2%) had liver cirrhosis and 369 (74.6%) had good liver function (Child-Pugh class A). Concerning the tumor characteristics, 74.3% of patients had a single tumor and 66.3% had a tumor size smaller than 3 cm, with most of the patients having BCLC stages 0 and A (88.4%). Patients were predominantly treated with radiofrequency ablation (64%), and a large proportion of patients were treated with a single ablation (88.6%).

**Table 1:** Baseline characteristic for the validation cohort, *n* = 483.

Characteristics	Values
**Age— no. (%)**	
≤60 years	273 (56.5)
>60 years	210 (43.5)
**Gender— no. (%)**	
Male	400(82.8)
Female	83 (17.2)
**Etiology— no. (%)**	
HBV	393(81.4)
HCV	60 (12.4)
ALD	30 (6.2)
Hypertension— no. (%)	151(31.3)
Diabetes— no. (%)	122(25.3)
Antiviral— no. (%)	298(61.7)
Smoking— no. (%)	221 (45.8)
Drinking— no. (%)	168(34.8)
Cirrhosis— no. (%)	431 (89.2)
**Child-Pugh class— no. (%)**	
A	369(76.4)
B	114 (23.6)
**Number of ablations — no. (%)**	
Single ablation	428(88.6)
Multiple ablations	55 (11.4)
**Ablative modality— no. (%)**	
RFA	309(64)
MWA	147 (30.4)
AHC	27 (5.6)
**AFP— no. (%)**	
<7 ng/ml	173 (35.8)
7–400 ng/ml	211 (43.7)
≥400 ng/ml	99 (20.5)
**DCP— no. (%)**	
<40 mAU/ml	308 (63.8)
≥40 mAU/ml	175 (36.2)
**BCLC stages— no. (%)**	
0	164(34)
A	263 (54.5)
B	56 (11.5)
**Tumor number — no. (%)**	
Single	359(74.3)
Multiple	124 (25.7)
**Tumor size — no. (%)**	
≤3 cm	320(66.3)
>3 cm	163 (33.7)
WBC (mean ± SD),10^9^/L	5.05 ± 1.97
RBC (mean ± SD),10^6^/L	4.21 ± 0.61
PLT (mean ± SD),10^9^/L	124.77 ± 62.45
ALT (mean ± SD), U/L	30.04 ± 16.54
AST (mean ± SD), U/L	30.32 ± 13.77
TBIL (mean ± SD), umol/L	19.68 ± 10.01
DBIL (mean ± SD), umol/L	8.11 ± 4.87
Total albumin (mean ± SD), g/L	66.18 ± 6.96
Globulin (mean ± SD), g/L,	28.99 ± 5.50
*γ*-GT (mean ± SD), U/L	64.01 ± 58.07
ALP (mean ± SD), U/L	87.71 ± 31.57
APR (mean ± SD),	0.25 ± 0.11
PT (mean ± SD), s	12.3 ± 1.51
INR (mean ± SD),	1.15 ± 0.13
Fibrinogen (mean ± SD), g/L	3.09 ± 0.98

HBV, hepatitis B virus; HCV, hepatitis C virus; ALD, alcohol liver disease; RFA, radiofrequency ablation; MWA, microwave ablation; AHC, argon-helium cryoablation; AFP, alpha-fetoprotein; DCP, des-γ-carboxyprothrombin; BCLC stages, Barcelona Clinic Liver Cancer stages; WBC, white blood cell; RBC, red blood cell; PLT, Platelet; ALT, alanine aminotransferase; AST, aspartate aminotransferase; TBIL, total bilirubin; DBIL, direct bilirubin; γ-GT, gamma-glutamyl transferase; ALP, alkaline phosphatase; APR, albumin-to-prealbumin ratio; PT, prothrombin time; INR, international normalized ratio; SD, standard deviation.

The median follow-up time for the validation cohort was 35.6 months, with a total of 292 (60.5%) patients experiencing recurrence by the end of follow-up, and the cumulative recurrence rates of 1, 2, and 3 years were 26.7% (129/483), 50.5% (244/483), and 58.0% (280/483), and the corresponding OS rate was 99.8% (482/483), 97.7% (472/483), and 93.8% (453/483), respectively.

There were no significant differences in baseline characteristics between the two cohorts by comparison with the historical data from the training cohort.

### Validation of the original scoring system in the validation cohort

**Figure 1:**
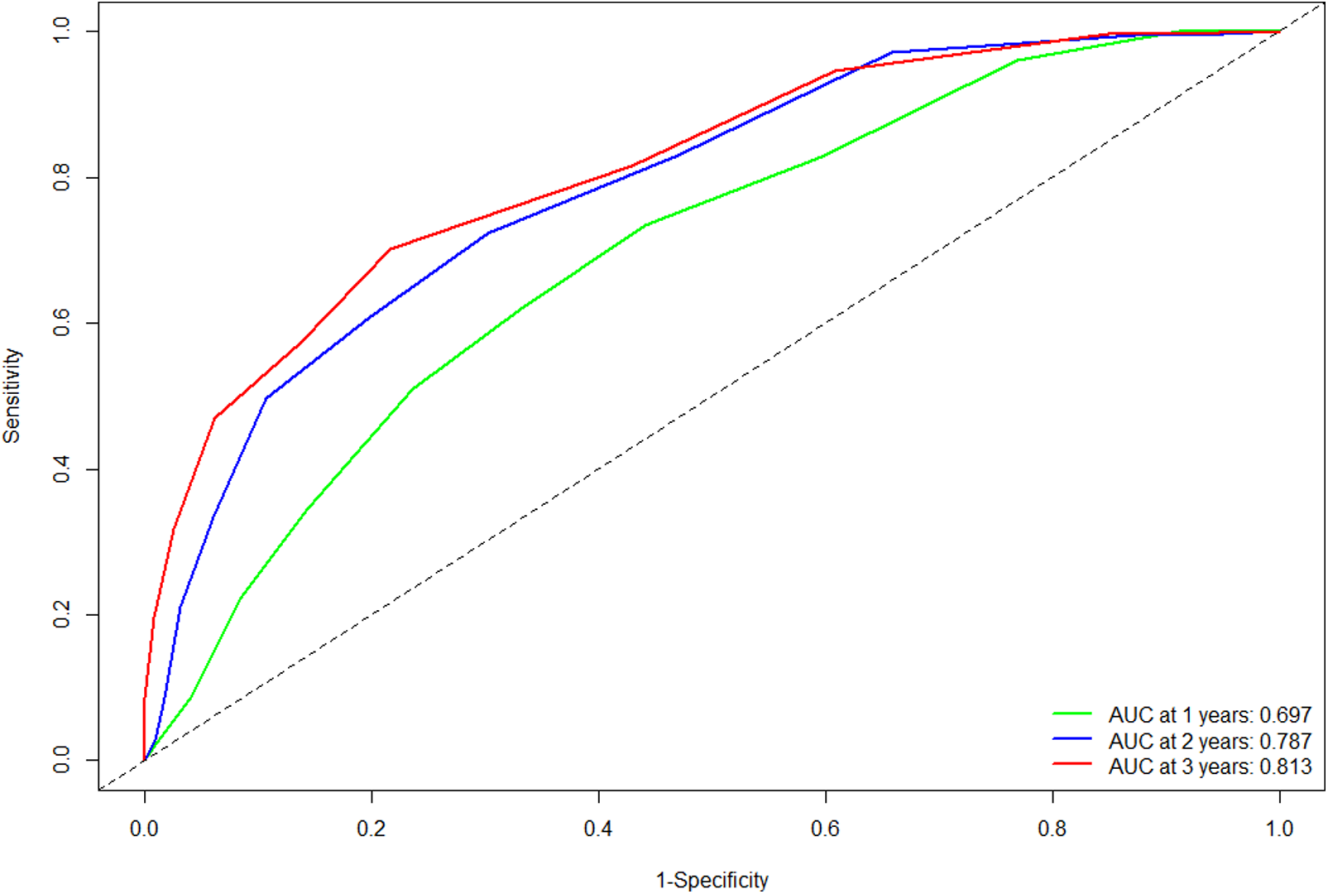
Comparison of the ROC curves of the original scoring system at different time points in the validation cohort. Abbreviations: ROC, receiver operating characteristics; AUC, area under the curve.

The C-statistic in the validation cohort was 0.695 [95% confidence interval (CI): 0.666- 0.724]. The AUCs of the time-dependent ROC curve were 0.680, 0.728, and 0.709 for 1-, 2-, and 3-year RFS in the training cohort (13). In the validation cohort, the AUCs at 1, 2, and 3 years were 0.697, 0.787, and 0.813, respectively (Figure 1). All the results suggested that the original scoring system has a good discriminatory ability for RFS in the validation cohort.

**Figure 2:**
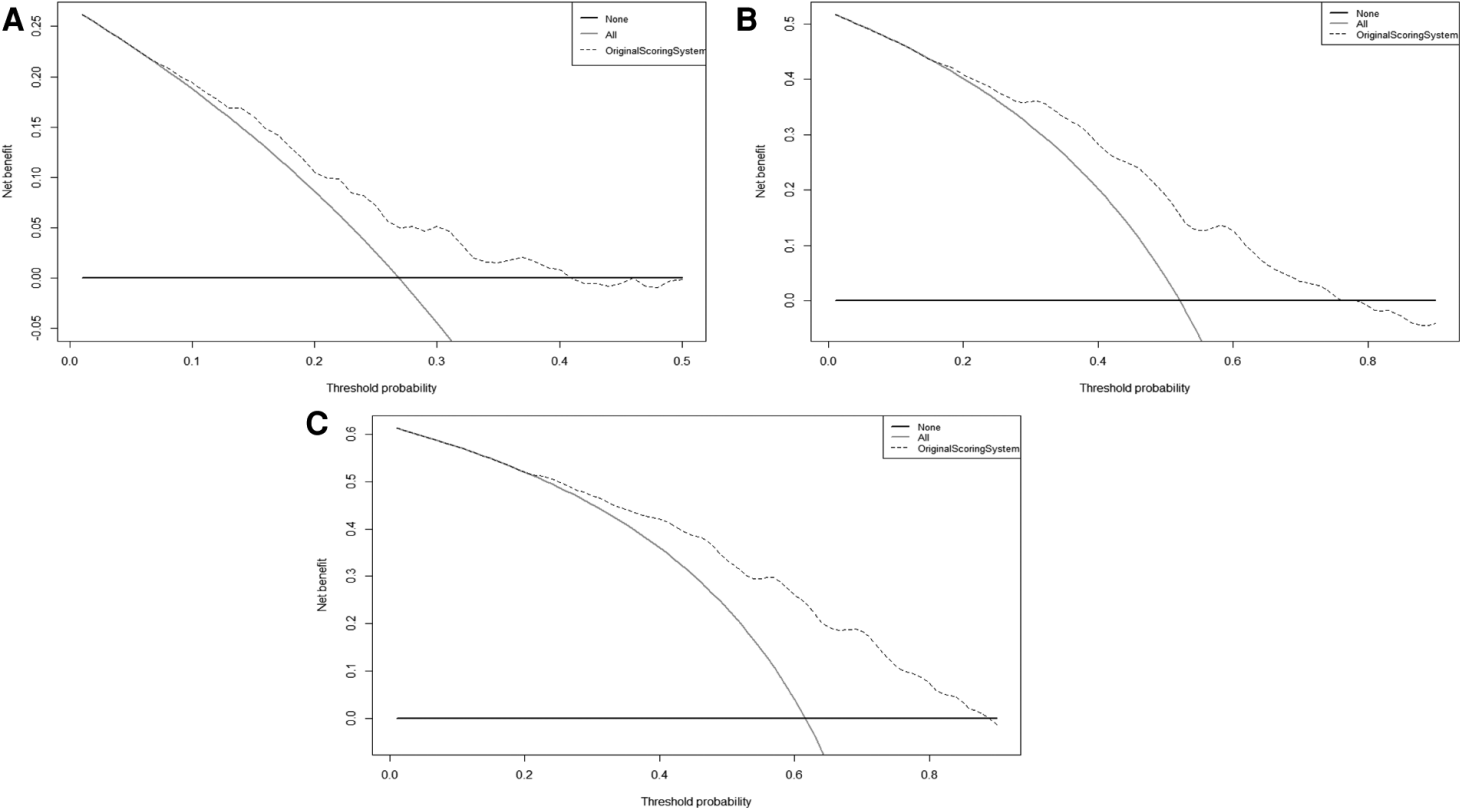
The DCA curves of original scoring system in 1(**A**), 2(**B**), and 3(**C**) years of RFS in the validation cohort. Abbreviations: RFS, recurrence-free survival; DCA, decision curve analysis.

Furthermore, the calibration plots showed an excellent agreement between the scoring system' predicted probability and observed probability for the 1-, 2- and 3-year in the validation cohort (Supplementary Figure S2). Also, the DCA plots showed that the scoring system had a favorable clinical net benefit in the validation cohort (Figure 2).

**Figure 3:**
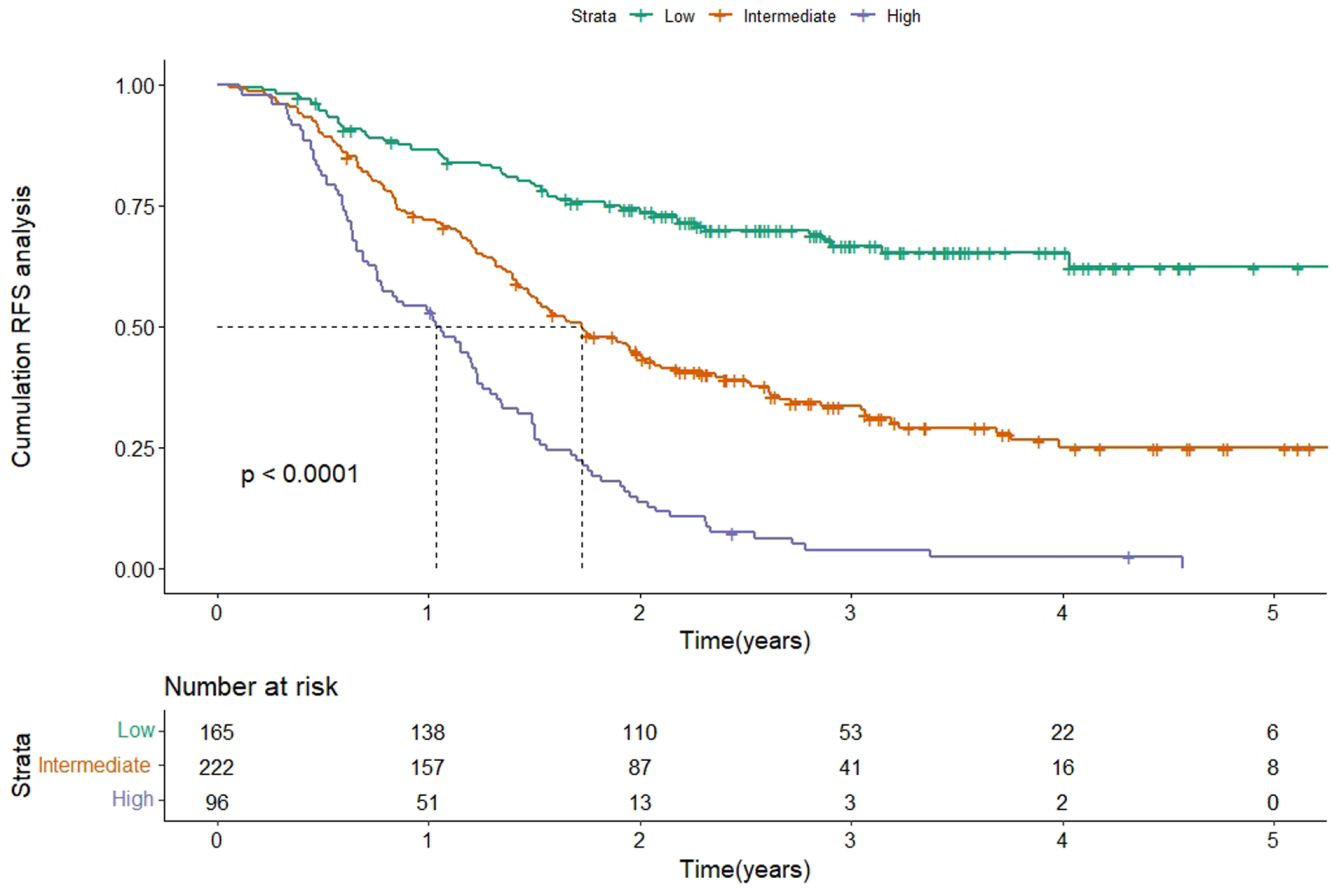
Kaplan-Meier curve of RFS according to the original scoring system in the validation cohort. Abbreviations: RFS, recurrence-free survival.

According to the scores of the original scoring system (13), the patients were divided into three groups: low-, intermediate-, and high-risk. Kaplan-Meier survival analysis was then performed on the RFS of the three groups. The results showed that the median RFS was 20.7 months (95% CI 17.4–24.1) and 12.4 months (95% CI 8.9–15.8) in the intermediate-risk and high-risk groups, and was not reached in the low-risk group (*P* < 0.001), which indicated a significant discriminatory ability of original scoring system for recurrence risks in the validation cohort (Figure 3).

### Improvement of the original scoring system (development of YA score)

Next, the scoring system was further optimized to improve the prediction performance. We reclassify the 2017–2019 cohort (the validation cohort mentioned above) into a new training cohort and a validation cohort, with the baseline information for both sets shown in (Table 2).

**Table 2:** Baseline characteristic for the training cohort and the validation cohort.

Characteristics	Training cohort (*N* = 355)	Validation cohort (*N* = 128)	*P* value
Age— no. (%)			0.892
≤60 years	200 (56.3)	73 (57.0)	
>60 years	155 (43.7)	55 (43.0)	
Gender— no. (%)			0.585
Male	292(82.3)	108 (84.4)	
Female	63 (17.7)	20 (15.6)	
Etiology— no. (%)			0.204
HBV	291(81.9)	102 (79.7)	
HCV	46 (13.0)	14 (10.9)	
ALD	18 (5.1)	12 (9.4)	
Hypertension— no. (%)	112(31.5)	39(30.5)	0.821
Diabetes— no. (%)	92(25.9)	30 (23.4)	0.580
Antiviral— no. (%)	219(61.7)	79(60.9)	0.995
Smoking— no. (%)	167 (47.0)	54 (42.2)	0.345
Drinking— no. (%)	127(35.8)	41(32.0)	0.446
Cirrhosis— no. (%)	317 (89.3)	114 (89.1)	0.942
Child-Pugh class— no. (%)			0.769
A	270 (76.1)	99 (77.3)	
B	85 (23.9)	29 (22.7)	
Number of ablations— no. (%)			0.138
Single ablation	310 (87.3)	118 (92.2)	
Multiple ablations	45 (12.7)	10 (7.8)	
Ablative modality— no. (%)			0.899
RFA	225 (63.4)	84 (65.6)	
MWA	110 (31.0)	37 (28.9)	
AHC	20 (5.6)	7 (5.5)	
AFP— no. (%)			0.177
<7 ng/ml	123 (34.7)	50 (39.1)	
7–400 ng/ml	152 (42.8)	59 (46.1)	
≥400 ng/ml	80 (22.5)	19 (14.8)	
DCP— no. (%)			0.228
<40 mAU/ml	232 (65.4)	76 (59.4)	
≥40 mAU/ml	123 (34.6)	52 (40.6)	
BCLC stages— no. (%)			0.170
0	114(32.1)	50 (39.0)	
A	195 (54.9)	68 (53.1)	
B	46 (13.0)	10 (7.9)	
Tumor number — no. (%)			0.064
Single	256 (72.1)	103 (80.5)	
Multiple	99 (27.9)	25 (19.5)	
Tumor size — no. (%)			0.794
≤3 cm	234(65.9)	86 (67.2)	
>3 cm	121 (34.1)	42 (32.8)	
WBC (mean ± SD),10^9^/L	5.10 ± 2.10	4.92 ± 1.58	0.302
RBC (mean ± SD),10^6^/L	4.20 ± 0.62	4.24 ± 0.59	0.475
PLT (mean ± SD),10^9^/L	122.73 ± 61.29	13.04 ± 65.47	0.231
ALT (mean ± SD), U/L	29.91 ± 15.46	30.41 ± 19.31	0.769
AST (mean ± SD), U/L	30.14 ± 11.73	30.82 ± 18.32	0.631
TBIL (mean ± SD), umol/L	19.79 ± 9.80	19.34 ± 10.57	0.657
DBIL (mean ± SD), umol/L	8.15 ± 4.68	7.97 ± 5.39	0.719
Total albumin (mean ± SD), g/L	66.09 ± 6.18	66.43 ± 8.79	0.638
Globulin (mean ± SD), g/L,	28.84 ± 5.35	29.39 ± 5.91	0.332
γ-GT (mean ± SD), U/L	65.33 ± 59.84	60.32 ± 52.94	0.404
ALP (mean ± SD), U/L	87.74 ± 31.52	87.61 ± 31.84	0.970
APR (mean ± SD),	0.25 ± 0.10	0.25 ± 0.13	0.552
PT (mean ± SD), s	12.89 ± 1.52	13.03 ± 1.46	0.386
INR (mean ± SD),	1.15 ± 0.13	1.16 ± 0.13	0.422
Fibrinogen (mean ± SD), g/L	3.09 ± 0.99	3.07 ± 0.94	0.869

RFA, radiofrequency ablation; MWA, microwave ablation; AHC, argon-helium cryoablation; HBV, hepatitis B virus; HCV, hepatitis C virus; ALD, alcohol liver disease; AFP, alpha-fetoprotein; ALT, alanine aminotransferase; AST, aspartate aminotransferase; γ-GT, gamma-glutamyltransferase; APR, albumin-to-prealbumin ratio, the APR was estimated as the albumin divided by the prealbumin.

Firstly, univariate analysis in the training cohort showed that gender, AFP, DCP, BCLC stage, tumor number, tumor size, globulin, DBIL, albumin-to-prealbumin ratio (APR), and fibrinogen (Fib) were significantly associated with RFS. These variables were then included in multivariate stepwise backward Cox regression and revealed that gender (HR = 1.692, 95% CI: 1.33–2.16, *P* < 0.001), AFP (HR = 1.234, 95% CI: 1.02–1.49, *P* = 0.027), DCP (HR = 1.336, 95%CI: 1.16–1.54, *P* < 0.001), tumor number (HR = 1.223, 95% CI: 1.06–1.41, *P* = 0.006), tumor size (HR = 1.331, 95%CI: 1.16–1.53, *P* < 0.001), Fib (HR = 1.029, 95% CI: 1.01–1.05, *P* = 0.019), and APR (HR = 3.46, 95% CI: 2.02–10.77, *P* < 0.001) were independent predictors of RFS (Table 3).

**Table 3:** Univariate and multivariate analysis of factors associated with RFS in the training cohort.

Variables	Univariate analysis			Multivariate analysis		
	HR	95%CI	*P* value	HR	95%CI	*P* value
Age	0.956	0.71–1.30	0.776			
Gender	1.71	1.32–2.21	**<0**.**001**	1.692	1.33–2.16	**<0**.**001**
Etiology	1.064	0.95–1.19	0.286			
Hypertension	1.098	0.80–1.51	0.563			
Diabetes	1.053	0.75–1.48	0.763			
Antiviral	0.949	0.69–1.30	0.746			
Smoking	0.979	0.72–1.34	0.893			
Drinking	1.071	0.78–1.47	0.672			
Cirrhosis	1.016	0.63–1.64	0.949			
Child-Pugh class	0.99	0.60–1.64	0.97			
Number of ablations	0.807	0.50–1.29	0.378			
Ablative modality	1.017	0.81–1.28	0.885			
AFP	1.31	1.07–1.61	**0**.**009**	1.234	1.02–1.49	**0**.**027**
DCP	1.365	1.17–1.59	**<0**.**001**	1.336	1.16–1.54	**<0**.**001**
BCLC stage	0.813	0.56–0.96	**0**.**02**	0.82	0.58–1.15	0.248
Tumor number	1.445	1.17–1.78	**0**.**001**	1.223	1.06–1.41	**0**.**006**
Tumor size	1.429	1.19–1.71	**<0**.**001**	1.331	1.16–1.53	**<0**.**001**
WBC	0.945	0.86–1.03	0.2			
RBC	0.863	0.62–1.19	0.374			
PLT	1	0.99–1.01	0.935			
ALT	0.999	0.98–1.01	0.91			
AST	0.995	0.97–1.01	0.557			
TBIL	1.018	0.98–1.04	0.216			
DBIL	0.945	0.91–0.99	**0**.**05**	0.999	0.97–1.03	0.96
Total albumin	1.001	0.95–1.04	0.954			
Globulin	1.018	1.01–1.07	**0**.**001**	1.244	0.93–1.67	0.145
γ-GT	1.001	0.99–1.01	0.664			
ALP	0.999	0.99–1.01	0.816			
APR	3.591	1.01–12.7	**0**.**048**	3.46	2.02–10.77	**<0**.**001**
PT	0.837	0.20-3.49	0.807			
INR	2.46	0.18–3.89	0.688			
Fibrinogen	1.401	1.01–1.94	**0**.**046**	1.029	1.01–1.05	**0**.**019**

AFP, alpha-fetoprotein; DCP, des-γ-carboxyprothrombin; BCLC stages, Barcelona Clinic Liver Cancer stages; WBC, white blood cell; RBC, red blood cell; PLT, Platelet; ALT, alanine aminotransferase; AST, aspartate aminotransferase; TBIL, total bilirubin; DBIL, direct bilirubin; γ-GT, gamma-glutamyl transferase; ALP, alkaline phosphatase; APR, albumin-to-prealbumin ratio; PT, prothrombin time; INR, international normalized ratio.

Based on the HR values of the above seven variables, a scoring system was obtained, which ranged from 0 to 14 by calculating the total score of included parameters (Table 4). Patients were re-separated equally according to their total score, with scores of 0–4 defined as low risk of recurrence, 5–9 as immediate risk of recurrence, and 10–14 as high risk of recurrence. The resulting score was named the YA score (the score of Beijing **Y**ou ‘**A**n Hospital).

**Table 4:** The YA score based on HR.

Variables	Scores
**Gender**	
Male	2
Female	0
**AFP (ng/ml)**	
<7	0
7–400	1
≥400	2
**DCP (mAU/ml)**	
<40 mAU/ml	0
≥40 mAU/ml	1
**Tumor number**	
Single	0
Multipl	2
**Tumor size**	
≤3 cm	0
>3 cm	2
**APR**	
<0.250	0
≥0.250	4
**Fibrinogen (mg/dl)**	
<3.105	0
≥3.105	1

AFP, alpha-fetoprotein; DCP, des-γ-carboxyprothrombin; APR, albumin-to-prealbumin ratio.

### Predictive performance of the YA score in the training cohort

**Figure 4:**
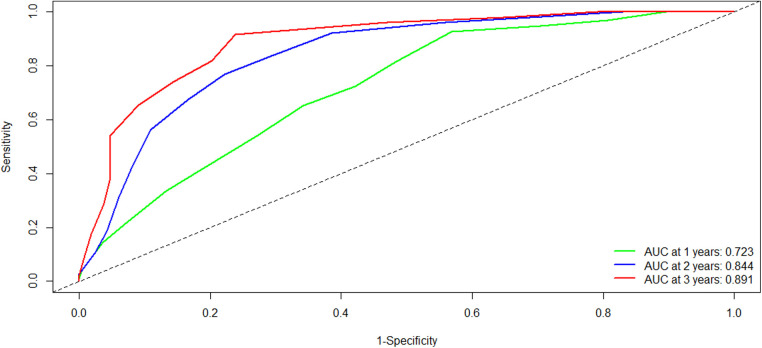
Comparison of the ROC curves of the YA score at different time points in the training cohort. Abbreviations: ROC, receiver operating characteristics; AUC, area under the curve.

To evaluate the discriminatory power of the YA score, we plotted the ROC curve and calculated the AUC in the training cohort. Firstly, the C-statistic for the YA score was 0.712 (95% CI: 0.675–0.749). As for the time-dependent AUCs of 1, 2, and 3 years, the YA scores were 0.723, 0.844, and 0.891(Figure 4), respectively, which were significantly better than the results of the original scoring system (0.697, 0.787 and 0.813), showing the prominence of discrimination in the YA score.

When comparing the Cox model fit with Kaplan-Meier plots, good agreement (calibration) between the predictions from the YA score to the observed probabilities was observed (Supplementary Figure S3). Meanwhile, the calibration plots in the YA score at 1, 2, and 3 years showed in Supplementary Figure S4 and also present an excellent agreement between the predicted probability and observed probability.

**Figure 5:**
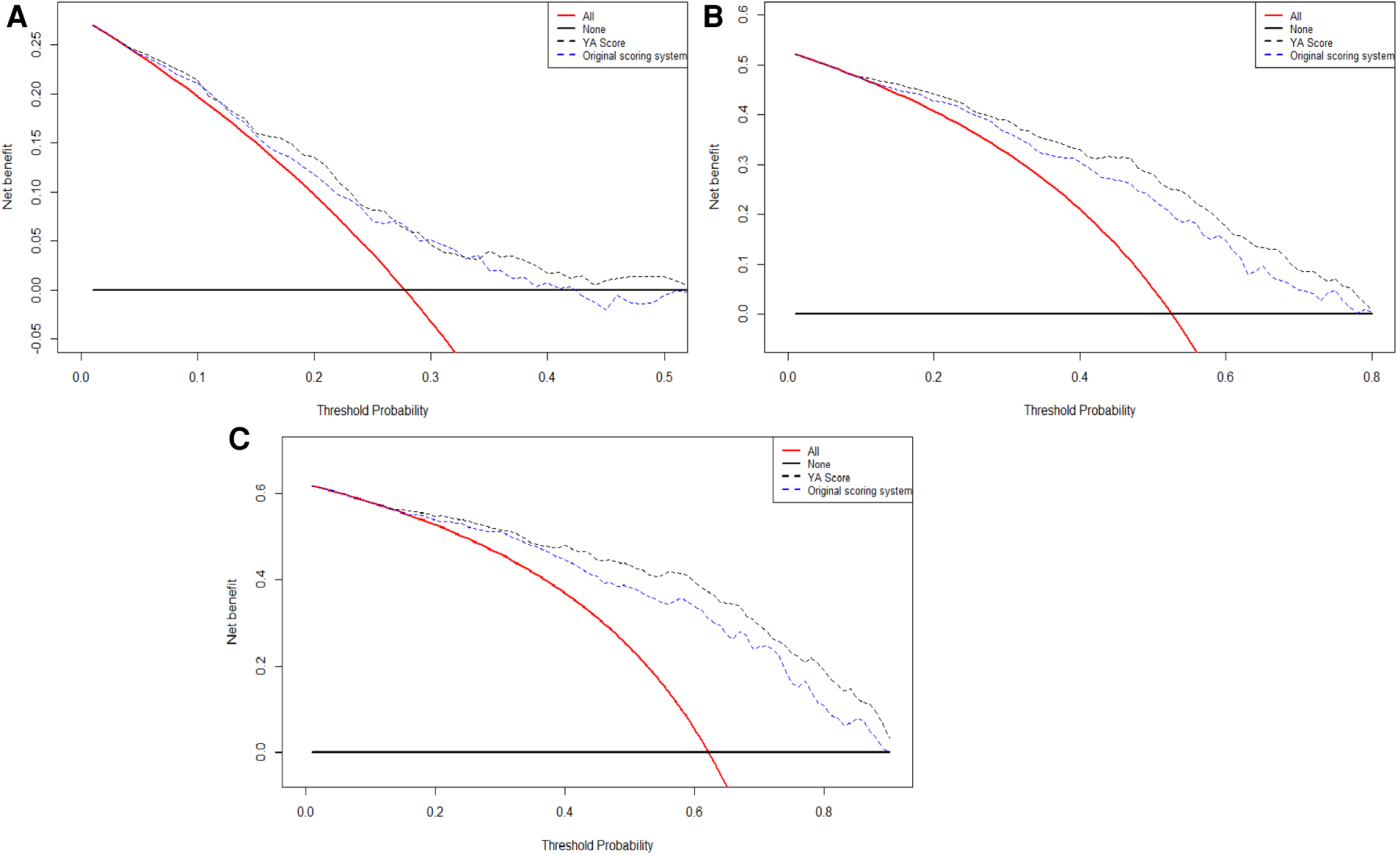
The DCA curves of the comparison between original scoring system and the YA score in 1(**A**), 2(**B**), and 3(**C**) years of RFS. Abbreviations: RFS, recurrence-free survival; DCA, decision curve analysis.

Lastly, the DCA curves suggested that using the YA score to predict RFS could increase the net benefit over the original scoring system (Figure 5).

Besides, the NRI was used to evaluate the improvement of risk prediction. The NRI of the 1-, 2- and 3-year was 0.276 (95% CI: 0.158–0.676), 0.682 (95% CI: 0.443–0.913), and 0.826 (95% CI: 0.657- 0.927), respectively, suggesting that the YA score has more significant potential for the correct prediction of recurrence compared to the original scoring system.

### Predictive performance of the YA score in the validation cohort

As for the time-dependent AUC at 1, 2, and 3 years in the validation cohort, the YA scores were 0.811, 0.847, and 0.902, respectively (Supplementary Figure S5), with a C-statistic of 0.787 (95% CI: 0.739–0.834). The calibration curves for the YA score demonstrated good agreement in the validation cohort (Supplementary Figure S6). And the DCA curves in the validation set al.so revealed that using YA scores to predict RFS can increment the net benefit (Supplementary Figure S7).

### Clinical application value of the YA score

**Figure 6:**
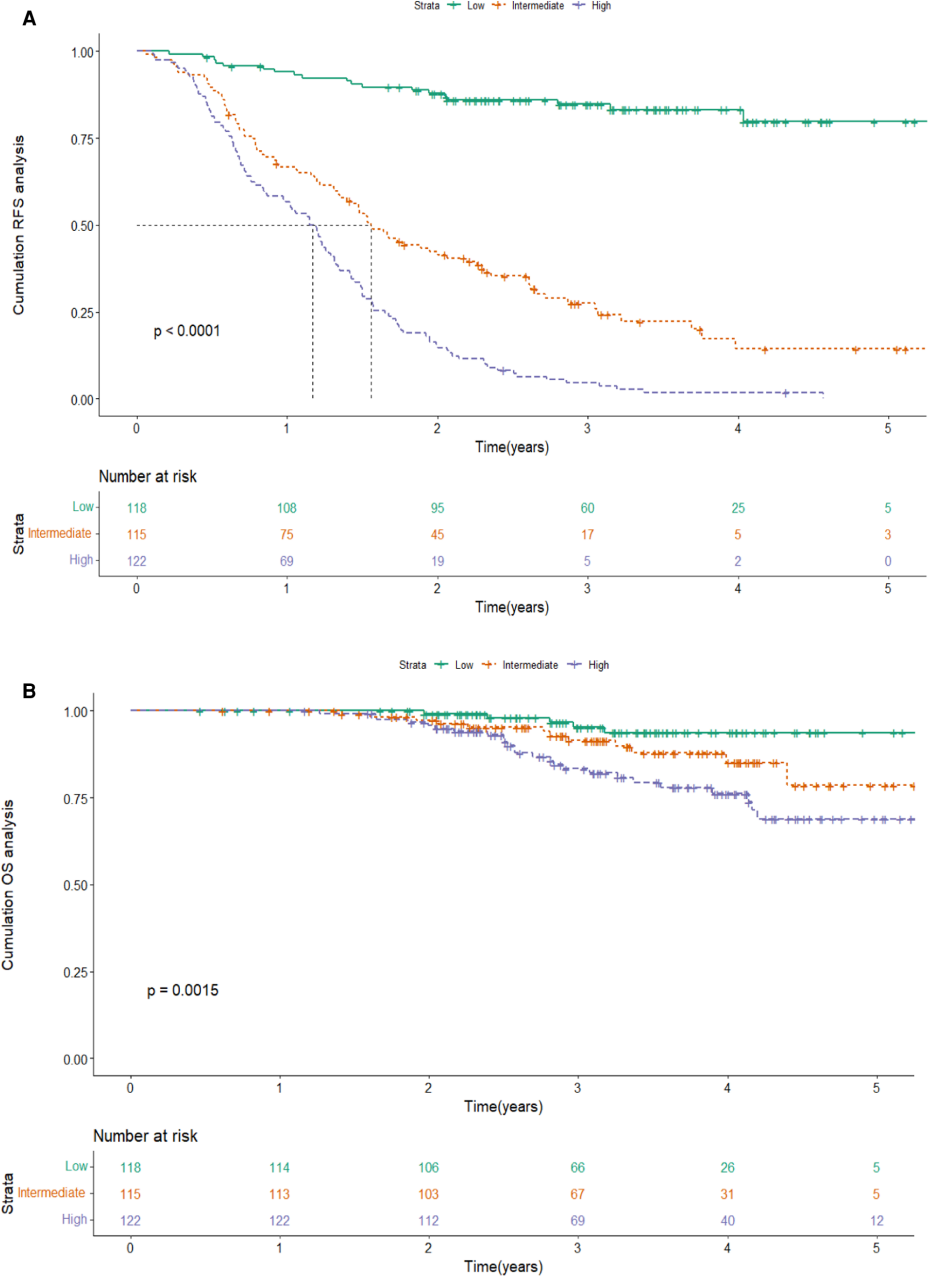
Kaplan-Meier curve of RFS(**A**) and OS (**B**) according to the YA score in the training cohort. Abbreviations: RFS, recurrence-free survival; OS, overall survival.

Based on the score of the YA score, the patients were divided into three groups. KM curves of RFS were then plotted, showing that the median RFS was 18.7 months (95% CI 15.7–21.7) and 13.8 months (95% CI 11.8–15.8) in the intermediate-risk and high-risk groups, and was not reached in the low-risk group. Note that, by the end of follow-up, half of the low-risk group had not yet relapsed, while about 50% of the high-risk group relapsed in the first year, which indicated a significant discriminatory ability for HCC patients at high risk for recurrence predicted by the YA score (*P* < 0.001) (Figure 6A). The YA score also has an excellent clinical application value for OS (Figure 6B), which was similar to the previous results (13).

In addition, the YA score provided better forewarning management of early relapse, with a C-statistic of 0.707 (95% CI: 0.668–0.746). And the calibration curves for the probability of 1- and 2-year RFS showed good agreement between prediction and observation in 181 early recurrence patients with HCC (Supplementary Figure S8).

### Comparison with other prognostic scores

We compared the predictive capacity of the YA score with those of five conventional prognostic scores. The outcomes suggested that the scoring system shows better discriminative power, which was markedly higher than the other five scores (Table 5).

**Table 5:** The AUROCs for predicting RFS of the YA score and other prognostic scores.

Prognostic scores	AUROC for RFS at 1 year	AUROC for RFS at 2 years	AUROC for RFS at 3 years
ALBI grade	0.527	0.534	0.541
PALBI grade	0.515	0.518	0.515
MLR	0.614	0.632	0.641
NLR	0.621	0.637	0.641
PLR	0.587	0.611	0.629
YA score	0.723	0.844	0.891

ALBI, albumin-bilirubin; PALBI, platelets - albumin – bilirubin; MLR, monocyte to lymphocyte ratio; NLR, neutrophil-lymphocyte ratio; PLR, platelet-lymphocyte ratio.

## Discussion

The original scoring system established in 2019 was used in a clinical trial to screen out HCC patients who were at high risk of relapse and then gave them anti-PD-1 immunotherapy after local treatment (TACE combined with ablation) to reduce the risk of recurrence. The results showed that the scoring system could stratify patients based on the different risks of relapse. Meanwhile, immunotherapy could effectively reduce the recurrence rate of HCC patients with high relapse risk predicted by our scoring system (19). Even though the scoring system had good clinical value, it was not validated externally due to the limitation of the number of cases at that time.

In this study, we performed temporal external validation of the original scoring system, and the results showed that it had good discrimination, with a C-index of 0.695. Meanwhile, the time-dependent AUC of 1 year, 2 years, and 3 years in the validation cohort were similar to the results in the training cohort. Also, the calibration curve and the DCA curve revealed that the original scoring system had high accuracy and positive net benefit, reconfirming the validity of the scoring system in predicting recurrence in HCC patients.

During the external validation process, we improved the original scoring system and finally developed a YA score based on seven variables. Additionally, the C-index of the YA score is better than the original scoring system (0.712 vs. 0.695), and the time-dependent AUC also shows significant superiority. Although the KM curves showed no significant difference between the two scores, the YA score could predict the recurrence of HCC patients more accurately by evaluating the NRI.

The occurrence and development of HCC is a complex process with numerous contributing factors. And diversity in tumor burden and liver function reserve exerts a crucial impact on the survival and clinical course of HCC (20). Thus, the predictive power of the scoring system could be further improved by giving a comprehensive evaluation of relevant variables (21). Yet for the convenience of clinical use, most models usually only included little clinical markers or radiological imaging outcomes, limiting the prediction effect of the model. In our study, the YA score with 7 risk variables covering tumor burden (tumor size and tumor number), serum tumor markers (AFP and DCP), liver function (APR), coagulation function (Fib), and gender, was established to dramatically enhance the predictive reliability. The other advantage of the YA score is that all parameters containing clinically available serologic markers and imaging results are easily accessible and contribute to clinical workup. Simultaneously, owing to the simplicity of the calculation, the patient's recurrence risk score can be comfortably calculated depending on the YA score, allowing clinical follow-up decisions to be made.

As we all know, the male gender has been a commonly recognized risk factor for HCC recurrence (22), and increasing evidence indicates that the prognosis of HCC may be related to gender disparity, with males having worse outcomes (23). Apart from that, indicators of liver functions are also associated with HCC, as abnormalities in liver function that persist may lead to inflammation, immune microenvironment disorder, and oxidative stress. In recent years, albumin and prealbumin, two serum biomarkers of liver function, have been demonstrated in several studies to be independent predictors of long-term prognosis for HCC (24). The APR was found to strongly predict recurrence after ablation in HCC patients in our previous study (13). In parallel, the HR of APR in the current study is 3.46, which remains an important variable for anticipating the risk of recurrence. Fibrinogen, an acute phase reactant produced by the liver in the presence of malignancy and/or systemic inflammation, is increased in patients with HCC, emerging as a novel predictor of clinical outcome (25).

Two new variables, tumor number, and DCP are added to the YA score. Contrary to our previous studies, the tumor number is an independent predictor of RFS in the current study, which could enhance the stability of the YA score for the reason that the combination of tumor number and tumor size could represent the tumor burden strongly correlated with recurrence after ablation in HCC patients, making it more effective to boost the predictability of YA score (26–28). Further, combined with other tumor markers could promote sensitivity, and specificity and make a reliable prognosis (29–31). The serum level of AFP correlated closely with tumor differentiation and aggressiveness (32), and was also a suggested indicator of hepatitis activity and severity, predicting the prognosis of HCC patients (33). DCP, another widely used highly specific diagnostic marker for HCC, could be a potentially potential predictor marker (34). In particular, DCP, having potential significance for the diagnosis of AFP-negative (35), may also be a prognostic supplement for AFP-negative patients. Our previous study was not evaluated for DCP for lack of validated data. In the present study, DCP is integrated into the YA score to strengthen the discriminatory ability of special populations.

As a first-line treatment for early-stage HCC, ablation therapy can produce comparable 5-year overall survival for HCC patients with early-stage compared to surgical resection (36, 37). While TACE is primarily recommended for patients with intermediate-stage, patients who cannot benefit from curative treatment, despite earlier-stage disease, could be good candidates for TACE (38). Many studies have shown that TACE combined with ablation has been shown to be more efficacious than either treatment alone (39, 40). However, few studies have examined prognostic markers in HCC patients treated with TACE and ablation, making the need for a scoring system in patient selection for combined (TACE + ablation) treatment increased. YA score, as a novel scoring system, can effectively predict the prognosis of patients after sequential treatment.

Compared with other HCC staging systems, the YA score is substantially outperformed in predicting recurrence. As well, our results also further reveal critical correlations between tumor burden, tumor markers, liver function, and early recurrence of HCC, providing useful perspectives for the exploration of early recurrence mechanisms. The outstanding predictive power facilitates the early detection of recurrent HCC, thereby reducing patient recurrence and improving the quality of life. Note that, patients were stratified into three subgroups according to the YA score, demonstrating that the YA score provides valid differentiation between patients with different risks of recurrence and death, which is favorable for the guidance of physicians in the close monitoring and adjuvant treatment.

Nevertheless, several limitations were associated with this study. Firstly, this study was a retrospective study conducted in a single center with selection bias, while a large number of cases and external validation over time enhance the generalization ability of the scoring system. Next, our scoring system was developed based on HCC patients of early-to-mid stage receiving local treatment, lacking the capability to predict the prognosis of patients with advanced HCC or patients treated with surgery or liver transplantation. With the improvement of medical treatment, however, a wider range of patients is being detected at an early stage, raising the prospect of the scoring system. Finally, our scoring system lacks external validation in other centers, requiring multi-center and large sample experiments for further analysis.

## Conclusion

In summary, by externally validating and improving the original scoring system, this study established the YA score, a novel, noninvasive, efficient, and feasible tool for predicting the postoperative prognosis of HCC patients after undergoing TACE plus ablation therapies, providing highly informative data for clinical management decisions.

## Data Availability

The original contributions presented in the study are included in the article/**Supplementary Material**, further inquiries can be directed to the corresponding author/s.
