# Supplementary figures and images for: External validation and improvement of the scoring system for predicting the prognosis in hepatocellular carcinoma after interventional therapy
Source: Front Surg. 2023 Mar 3;10:1045213. doi: 10.3389/fsurg.2023.1045213 (PMC10020369; doi:10.3389/fsurg.2023.1045213)

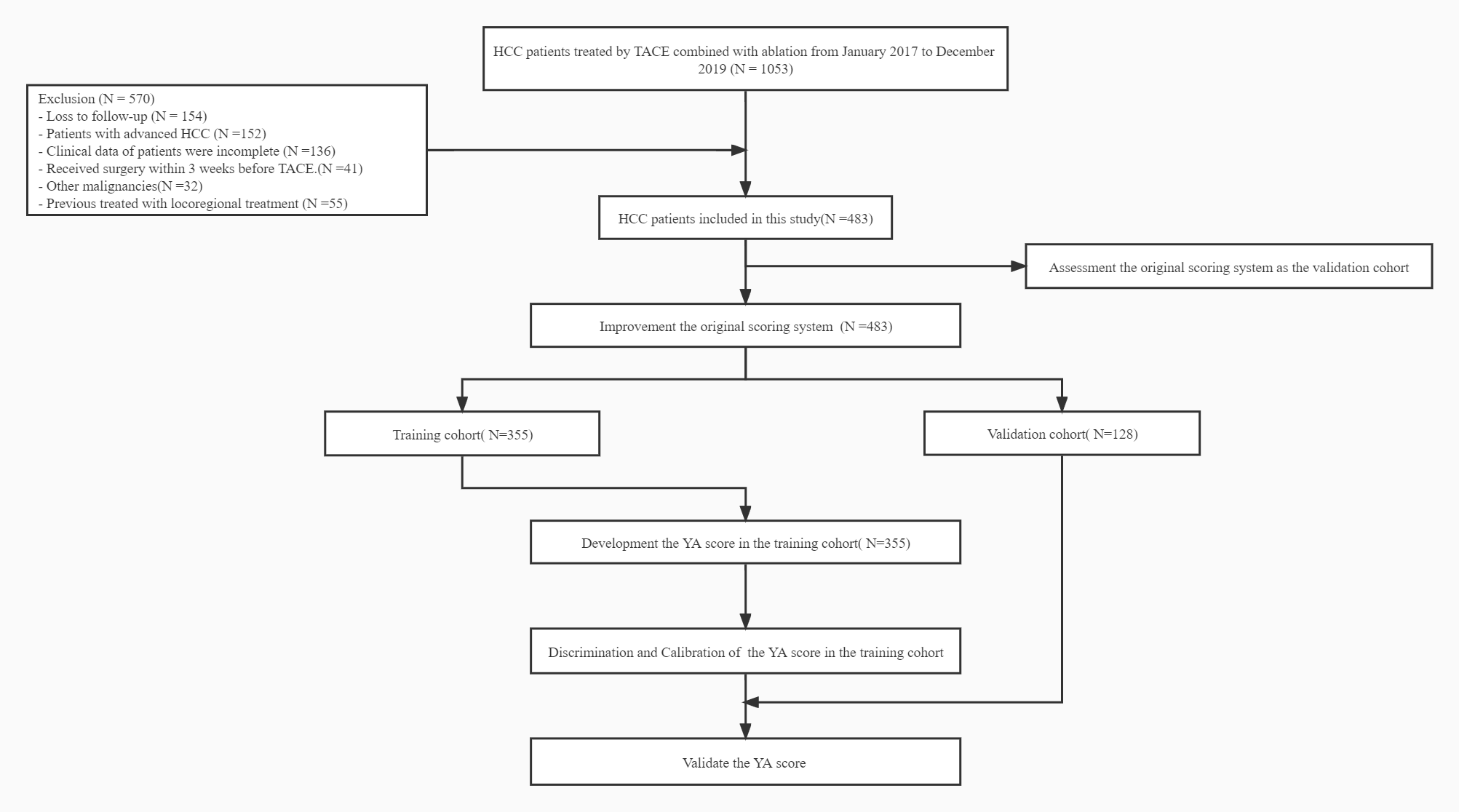

Supplement: Supplementary file 1 [file Image1.jpeg]

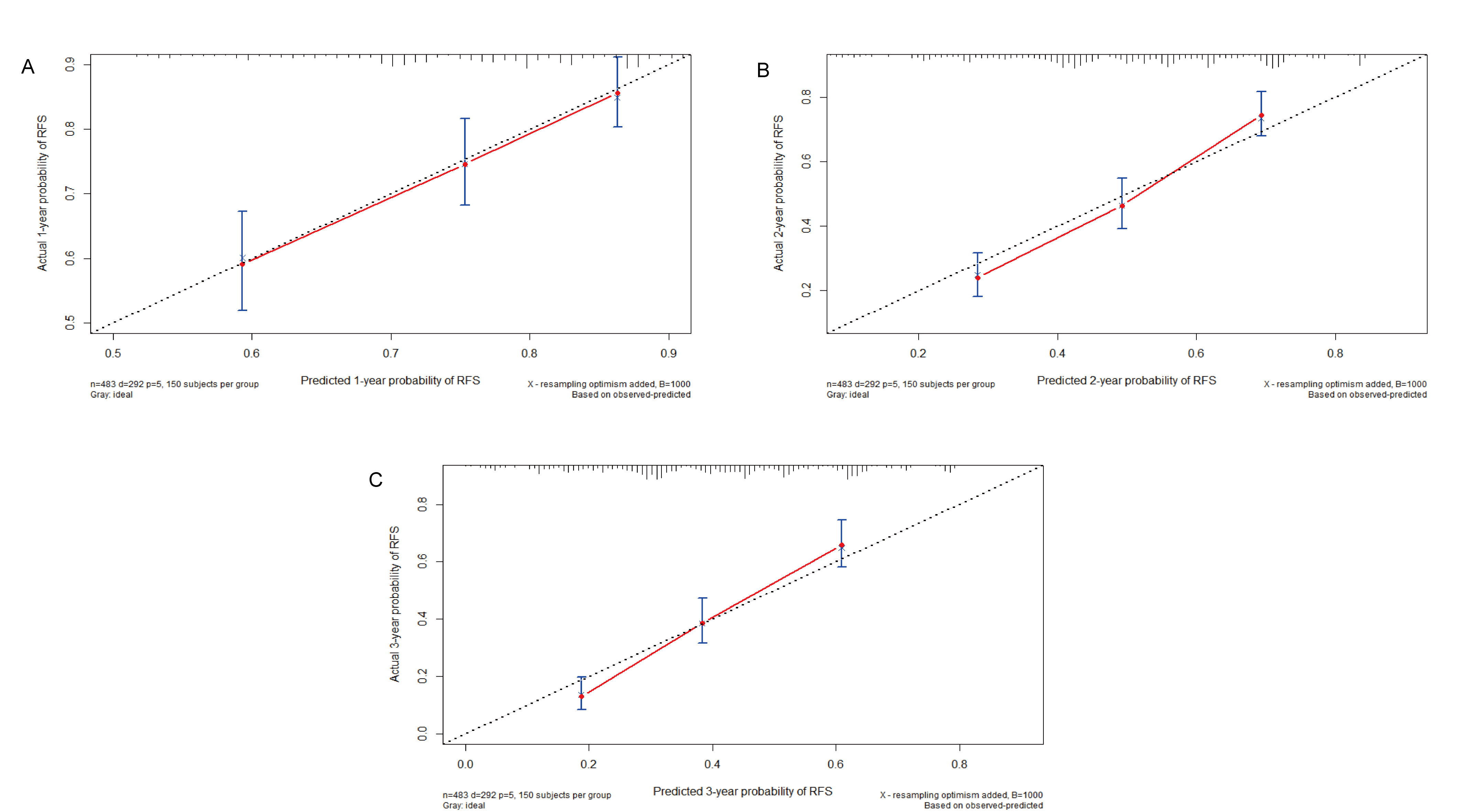

Supplement: Supplementary file 2 [file Image2.tif]

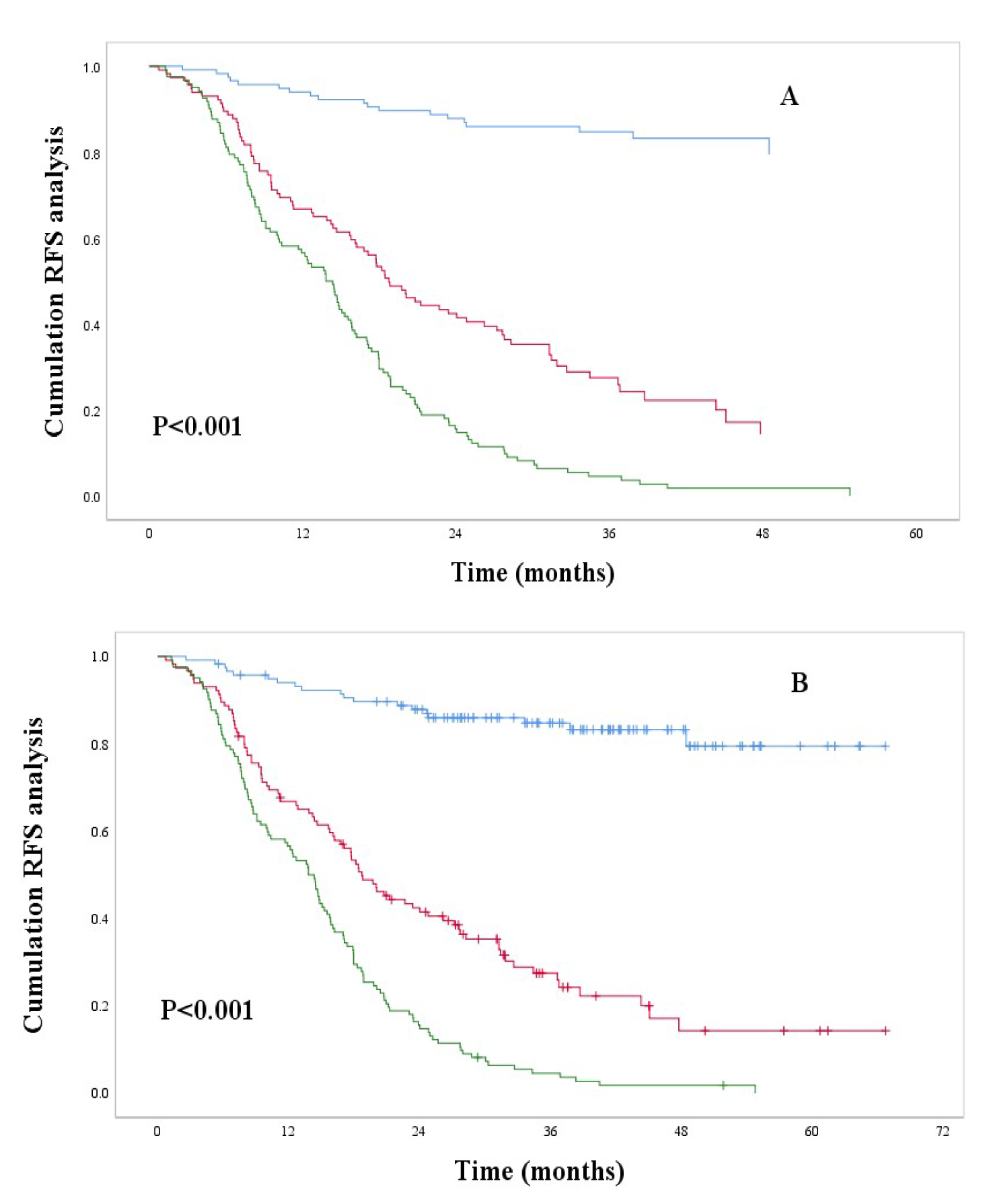

Supplement: Supplementary file 3 [file Image3.tif]

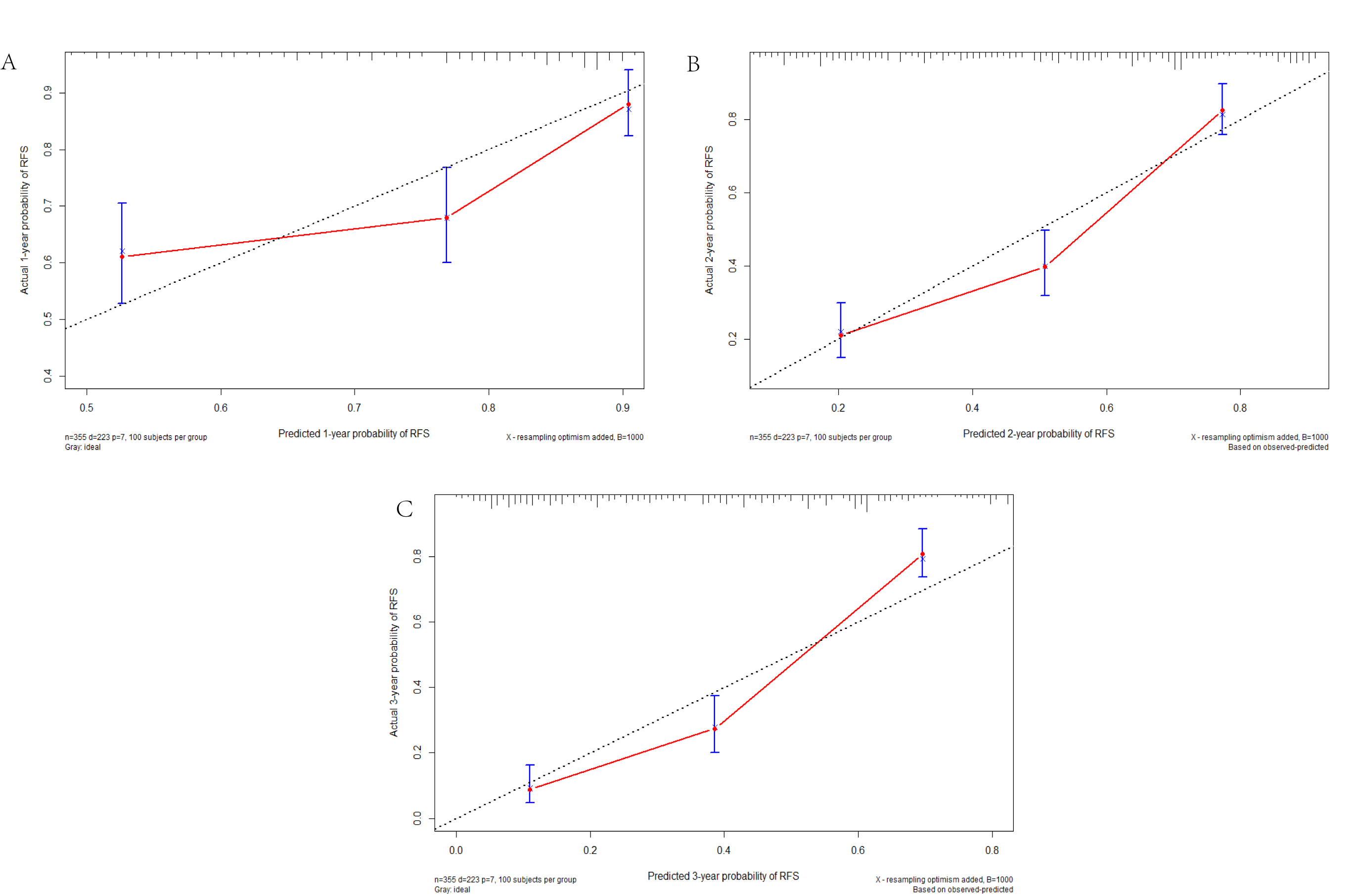

Supplement: Supplementary file 4 [file Image4.tif]

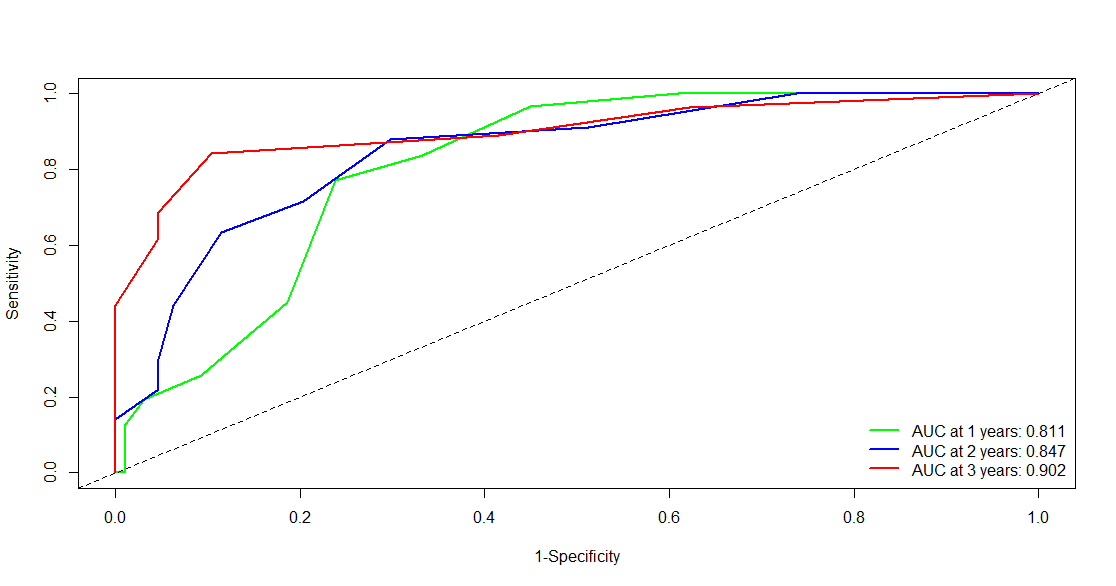

Supplement: Supplementary file 5 [file Image5.tiff]

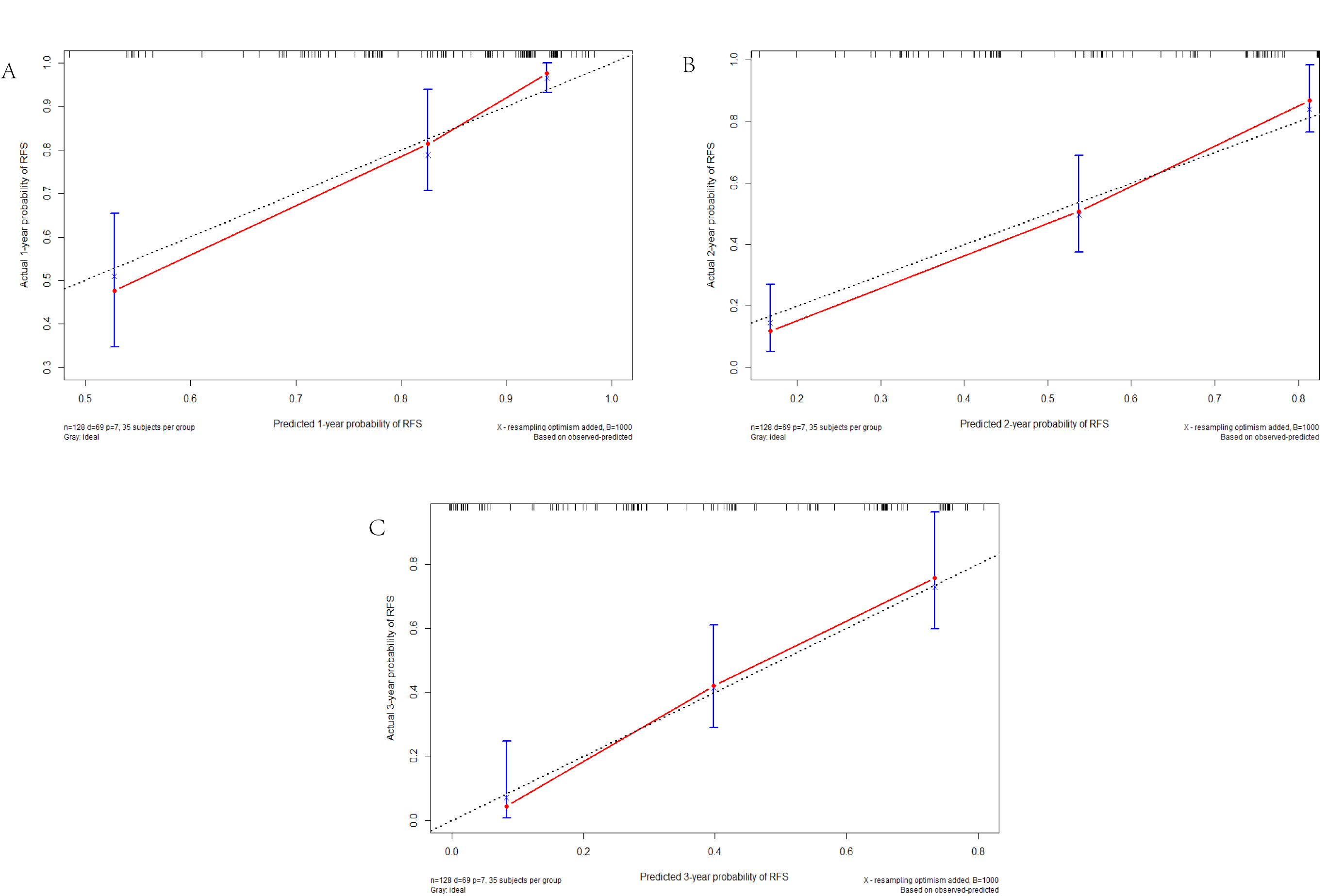

Supplement: Supplementary file 6 [file Image6.tif]

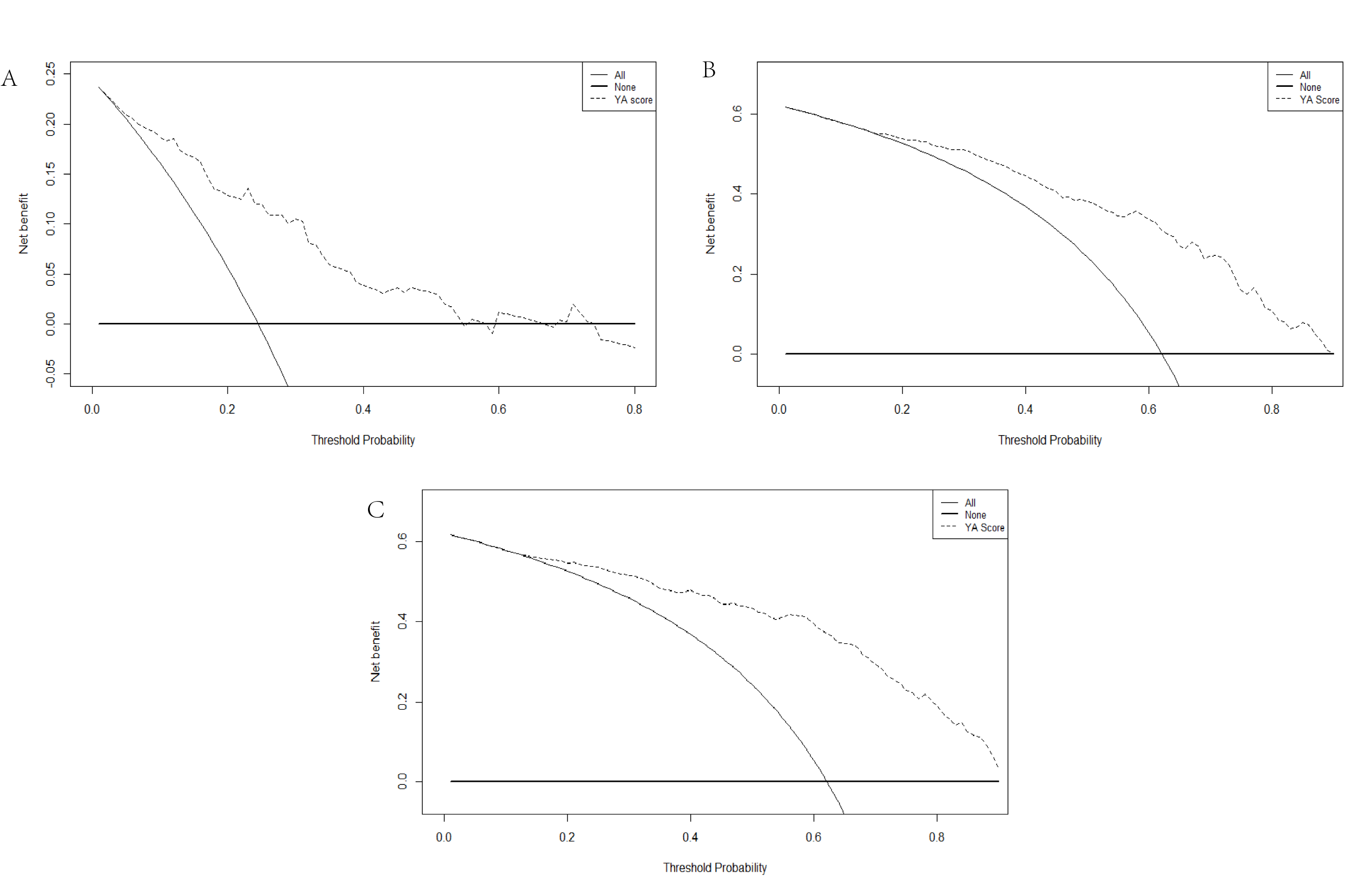

Supplement: Supplementary file 7 [file Image7.tif]

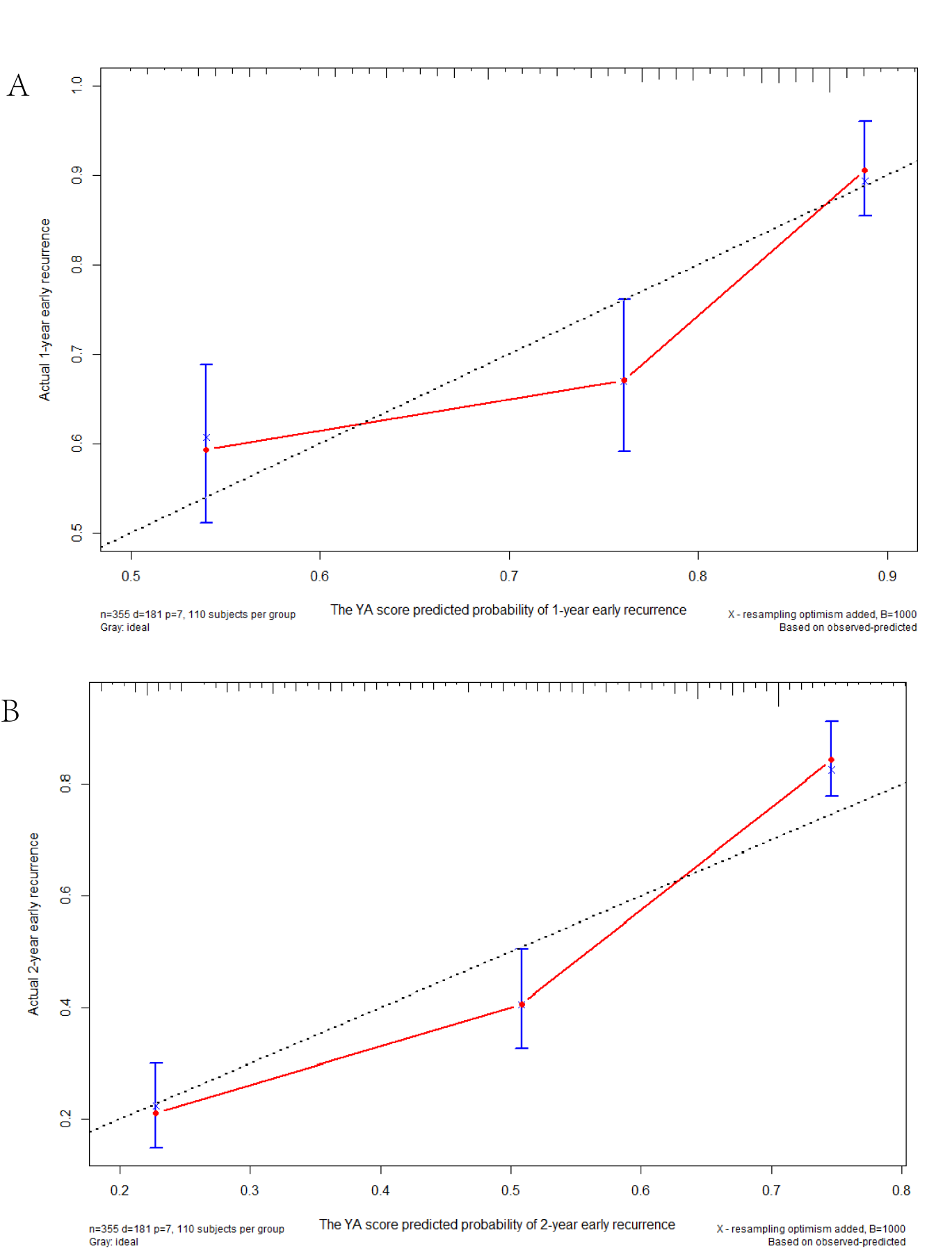

Supplement: Supplementary file 8 [file Image8.tif]
